# Evaluation of Alectinib Metabolic Stability in HLMs Using Fast LC-MS/MS Method: In Silico ADME Profile, P450 Metabolic Lability, and Toxic Alerts Screening

**DOI:** 10.3390/pharmaceutics15102449

**Published:** 2023-10-11

**Authors:** Mohamed W. Attwa, Haitham AlRabiah, Gamal A. E. Mostafa, Adnan A. Kadi

**Affiliations:** Department of Pharmaceutical Chemistry, College of Pharmacy, King Saud University, P.O. Box 2457, Riyadh 11451, Saudi Arabia; halrabiah@ksu.edu.sa (H.A.); gmostafa@ksu.edu.sa (G.A.E.M.); akadi@ksu.edu.sa (A.A.K.)

**Keywords:** alectinib, in vitro half-life, intrinsic clearance, ADME profile, metabolic stability, greenness, P450 metabolic mode, DEREK software, LC-MS/MS approach, StarDrop software

## Abstract

Alectinib, also known as Alecensa^®^, is prescribed for the therapeutic treatment of individuals diagnosed with metastatic non-small cell lung cancer (NSCLC) who have a specific genetic mutation referred to as anaplastic lymphoma kinase (ALK) positivity. The Food and Drug Administration granted regular approval to alectinib, a drug developed by Hoffmann-La Roche, Inc. (Basel, Switzerland)/Genentech, Inc. (South San Francisco, CA, USA), on 6 November 2017. The screening of the metabolic stability and identification of hazardous alarms within the chemical structure of ALC was conducted using the StarDrop software package (version 6.6), which incorporates the P450 metabolic module and DEREK software (KB 2018 1.1). The primary aim of this investigation was to develop a high-throughput and accurate LC-MS/MS technique for the quantification of ALC in the metabolic matrix (human liver microsomes; HLMs). The aforementioned methodology was subsequently employed to assess the metabolic stability of ALC in HLMs through in vitro tests, with the obtained results further validated using in silico software. The calibration curve of the ALC showed a linear correlation that exists within the concentration range from 1 to 3000 ng/mL. The LC-MS/MS approach that was recommended exhibited accuracy and precision levels for both inter-day and intra-day measurements. Specifically, the accuracy values ranged from −2.56% to 3.45%, while the precision values ranged from −3.78% to 4.33%. The sensitivity of the established approach was proved by its ability to adhere to an LLOQ of 0.82 ng/mL. The half-life (t_1/2_) and intrinsic clearance (Cl_int_) of ALC were estimated to be 22.28 min and 36.37 mL/min/kg, correspondingly, using in vitro experiments. The ALC exhibited a moderate extraction ratio. The metabolic stability and safety properties of newly created derivatives can be enhanced by making modest adjustments to the morpholine and piperidine rings or by substituting the substituent, as per computational software. In in silico ADME prediction, ALC was shown to have poor water solubility and high gastrointestinal absorption along with inhibition of some cytochrome P450s (CYP2C19 and CYP2C9) without inhibition of others (CYP1A2, CYP3A4, and CYP2D6) and P-glycoprotein substrate. The study design that involves using both laboratory experiments and different in silico software demonstrates a novel and groundbreaking approach in the establishment and uniformization of LC-MS/MS techniques for the estimation of ALC concentrations, identifying structural alerts and the assessment of its metabolic stability. The utilization of this study strategy has the potential to be employed in the screening and optimization of prospective compounds during the drug creation process. This strategy may also facilitate the development of novel derivatives of the medicine that maintain the same biological action by targeted structural modifications, based on an understanding of the structural alerts included within the chemical structure of ALC.

## 1. Introduction

Cancer is widely recognized as the primary global cause of mortality, characterized by uncontrolled cellular proliferation within a specific organ of the human body. Additionally, it has the potential to metastasize to other organs, attributed to genetic abnormalities that compromise the regulation of diverse cellular processes, thereby facilitating the development of malignant cells [1]. Over the past twenty years, a significant number of tyrosine kinase inhibitors (TKIs) have been created and received market approval for therapeutic intervention in diverse hematological malignancies and solid tumors [2]. The management of cancer has been facilitated through the utilization of molecular targeting strategies, which involve the regulation of suppressor genes and tumor oncogenes that play a role in the development of human cancer [3].

Lung cancer is a highly prevalent and lethal form of malignancy that is widely observed across the globe. Non-small cell lung cancers (NSCLCs) account for approximately 90% of all lung malignancies and encompass a variety of subtypes that arise from the activation of distinct oncogenes [4,5]. The initial characterization of rearrangements involving the anaplastic lymphoma kinase (ALK+)-activating gene occurred in individuals diagnosed with anaplastic large cell lymphoma (ALCL). Subsequently, these rearrangements have been identified as oncogenic drivers in a subset of patients (about 3–7%) with NSCLC as well as other types of cancer [6]. Following this, the rearrangement of ALK is regarded as a highly promising molecular target for therapeutic intervention.

In the preceding decade, the scientific community has witnessed the development of three successive cohorts of ALK inhibitors. These cohorts encompass the first-generation inhibitors, represented by crizotinib, the second-generation inhibitors, which include brigatinib, alectinib, ceritinib, and ensartinib, and finally, the third-generation inhibitors, exemplified by lorlatinib [7]. Crizotinib, a first-generation ALK inhibitor, is recognized as a multi-targeted tyrosine kinase inhibitor (TKI) due to its ability to inhibit not just ALK but also MET and ROS1. Some patients may demonstrate intolerance to the first-generation ALK inhibitor, crizotinib, and often experience the development of resistance. As a result, there has been the development of new versions of ALK inhibitors. Alectinib (ALC) has been recognized as the prevailing initial therapy for patients diagnosed with advanced NSCLC who possess ALK gene alterations, particularly in cases where resistance emerges and subsequently results in clinical relapse. Lorlatinib exhibits a high degree of central nervous system (CNS) penetration and has exhibited significant efficacy within the brain, even in individuals who have previously experienced treatment failure with a brain-penetrable TKIs like ALC [2].

**Figure 1:**
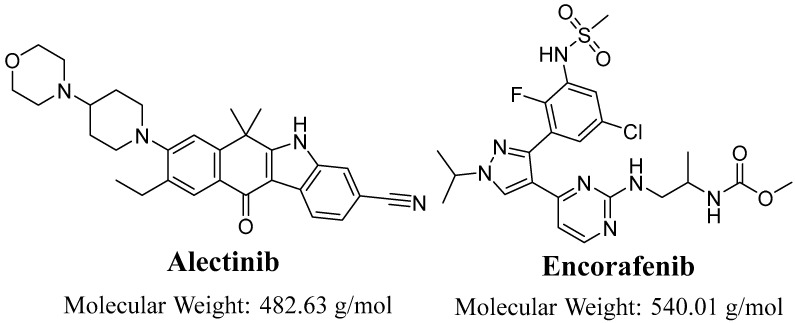
Chemical structure of alectinib and encorafenib (IS).

Alectinib (ALC; Figure 1), also known as CH5424802, is a pharmaceutical compound classified as a tyrosine kinase inhibitor. Its primary targets are the ALK and ret proto-oncogene (RET) kinases. ALC has been identified as a potent second-generation ALK inhibitor, demonstrating efficacy against a wide range of ALK mutations. ALC, known by its brand name Alecensa^®^, has received approval from both the US Food and Drug Administration (FDA) and the European Medicines Agency (EMA) for its use as a first-line treatment in patients with ALK-positive metastatic non-small cell lung cancer (ALK+ NSCLC) who have developed resistance to crizotinib or whose disease has progressed despite treatment with crizotinib [8,9]. The prevailing adverse symptoms associated with ALC included myalgia (29%), edema (30%), constipation (34%), and fatigue (41%). Dose reductions and treatment interruptions due to adverse events occurred in 12% and 27% of patients receiving alectinib, respectively. In contrast, a mere 6% of patients experienced the discontinuation of alectinib treatment due to adverse effects [8,10]. Despite the presence of previously documented side effects, it seems that the safety profile of ALC is comparatively more advantageous when compared to other ALK inhibitors. The administration of crizotinib to patients with ALK-rearranged NSCLC was found to be correlated with an increased incidence of visual impairments, gastrointestinal toxicities, and pneumonitis. However, it is worth noting that the majority of these adverse events were classified as grade 1 and 2. This can be attributed to the higher selectivity of ALC in inhibiting ALK, as compared to crizotinib, which also inhibits other significant kinases such as ROS1 and MET [11].

It is imperative to establish a rapid, environmentally friendly, and precise LC-MS/MS technique for the determination of ALC in diverse matrices. After conducting a comprehensive review of the available literature, it was found that no published articles exist about the quantification of ALC in HLMs, specifically in relation to the assessment of its metabolic stability. Therefore, the current study aimed to establish a highly sensitive (1 ng), quick (2 min), and specific liquid chromatography–tandem mass spectrometry (LC-MS/MS) approach for evaluating the metabolic stability of ALC in human liver microsome (HLM) matrix. Additionally, the obtained results were validated utilizing in silico analysis through the utilization of StarDrop software (version 6.6) and in vitro experiments using metabolic incubations.

The LC-MS/MS method utilized mobile phase in an isocratic system with a running duration of 2 min, characterized by its rapidity. The flow rate of the mobile phase was set at 0.5 mL, with a lower concentration of acetonitrile (55%) to promote environmental sustainability (greenness of the analytical method). In recent times, there has been a growing focus on green analytical chemistry (GAC), a field that seeks to mitigate the presence of hazardous chemicals, reduce energy consumption, and minimize waste generation through a range of analytical procedures [12,13]. In order to accomplish these aims, many measuring methodologies have been utilized to estimate the greenness of different analytical findings. The tactics encompassed in this set consist of the Analytical Eco-Scale (AES), National Environmental Methods Index (NEMI), Red–Green–Blue (RGB), Green Analytical Procedures Index (GAPI), and Analytical Greenness Metric Approach (AGREE) [12]. The approaches AES, GAPI, NEMI, and RGB exhibited a reliance on certain values of GAC, as illustrated. The “AGREE” methodology was employed to measure the level of environmental sustainability by evaluating twelve Greenness Assessment Criteria (GAC) and assigning them corresponding scores.

The method demonstrated a linear range spanning from 1 ng/mL to 3000 ng/mL, indicating its suitability for quantitative analysis. The confirmation of ALC metabolic stability assessment was achieved by evaluating the in silico ALC metabolic lability using the in silico P450 model provided by StarDrop software. This evaluation was conducted prior to initiating the in vitro metabolic incubations and the creation of the analytical method. The purpose of this approach was to optimize time and financial resources [14]. The LC-MS/MS methodology was utilized to evaluate the in vitro half-life (t_1/2_) and intrinsic clearance (Cl_int_) of ALC [15]. These parameters can be utilized to estimate the in vivo metabolic rate through the implementation of three models: parallel tube, venous equilibrium, and dispersion [16,17]. The determination of the half-life (t_1/2_) and clearance (Cl_int_) of ALC was conducted through the utilization of an in vitro methodology referred to as the “well-stirred” model [16,17]. The utilization of this model is frequently observed in drug metabolism investigations owing to its inherent simplicity. The metabolic rate of ALC was determined to be moderate, indicating a favorable level of in vivo bioavailability and a moderate duration of action, as evidenced by previous studies [18,19,20]. A computational analysis was performed to determine the absorption, distribution, metabolism, and excretion (ADME) profile of ALC, in order to anticipate its drug-like properties.

## 2. Materials and Methods

### 2.1. Materials

The solvents used in the current investigation, specifically ACN and H_2_O, were of HPLC quality. The reference powders utilized in this investigation, specifically alectinib and encorafenib, along with the solid chemicals employed, were of analytical grade (AR). The two target analytes, namely alectinib (catalog number: HY-13011, purity: 99.88%) and encorafenib (catalog number: HY-15605, purity: 99.63%), were obtained from MedChem corporation, which is situated in Princeton, NJ, USA. The chemicals ammonium acetate (CH_3_COONH_4_), formic acid, HLMs, and ACN used in this study were obtained from Sigma-Aldrich, a business headquartered in St. Louis, MO, USA. The HLMs at a concentration of 20 mg/mL were stored in a refrigerator at −78 °C upon arrival, using dry ice as a cooling agent, until they were prepared for utilization.

### 2.2. Instruments

The LC-MS/MS equipment used in this investigation comprised an Acquity UPLC (H10UPH) and an Acquity TQD MS (QBB1203). The utilization of this system was implemented in order to achieve chromatographic separation, mass analysis, and quantification of the target analytes (ALC and EFB) following extracting from the metabolic incubation (HLMs). The LC-MS/MS apparatus was utilized with the MassLynx software package (version 4.1, SCN 805). The process of data analysis was executed, and the outcomes were assessed utilizing the QuanLynx program (version 4.1, SCN 805).

The vacuum required for the TQD analyzer was achieved by employing a vacuum pump obtained from Sogevac, a Murrysville-based firm in the United States (New Stanton, PA, USA). The tuning of the properties of MS for ALC and EFB (IS) was carried out utilizing the IntelliStart^®^ application. The nitrogen gas utilized as the desiccating agent for the mobile phase in the electrospray source (ESI) system was produced by a nitrogen generator procured from Peak Scientific, a Scotland-based company in Inchinnan, UK. The collision cell of the TQD analyzer was used to fragment the parent ions of ALC and EFB into their respective daughter ions. This fragmentation process was achieved by introducing argon gas (99.999% purity) as the collision gas. The HPLC grade water employed in this investigation was produced utilizing an in-house Milli-Q water purification system developed by Millipore Corporation, situated in Billerica, MA, USA.

### 2.3. In Silico Assessment of ALC Metabolic Stability

The assessment of the metabolic lability of ALC was conducted using the P450 module of the StarDrop software developed by Optibrium Ltd., in Cambridge, MA, USA. This assessment was conducted before pursuing the in vitro incubation of ALC with HLMs. The confirmation of the importance of carrying out in vitro incubations and developing the LC-MS/MS method was achieved through the utilization of the results and data generated from the StarDrop software program. The findings were reported in the form of a composite site lability (CSL), which serves as an indicator of the metabolic stability of ALC [21].

In order to determine the ALC CSL, the SMILES representation of the compound (CCc1cc2c(cc1N3CCC(CC3)N4CCOCC4)C(c5c(c6ccc(cc6[nH]5)C#N)C2=O)(C)C) was entered into the StarDrop software. To assess the metabolic stability of ALC, we computed the reactivity of its constituent atoms and subsequently aggregated these values to derive the CSL value. The aforementioned value was then employed to determine the metabolic lability of ALC [22,23]. The CSL was calculated using Equation (1).

(1) CSL=ktotal(ktotal+kw)

where k_w_ is the rate constant for water formation.

### 2.4. In Silico Screening of the Toxicity of ALC Using DEREK Software

The assessment of potential toxicity for ALC was conducted using the DEREK module (KB 2018 1.1) of the StarDrop package (version 6.6). Furthermore, the program was employed to identify structural manifestations associated with ALC, with the aim of suggesting structural alterations that may potentially mitigate the observed toxicity [24,25].

### 2.5. In Silico ADME Analysis

The SwissADME software, version 1, developed by the Swiss Institute of Bioinformatics in Lausanne, Switzerland, was utilized to predict the absorption, distribution, metabolism, and excretion (ADME) properties of ALC. The software under consideration can be accessed through the web platform located at http://www.swissadme.ch/. The date of access for this software was 20 August 2023.

### 2.6. LC-MS/MS Instrumental Characteristics

The LC-MS/MS system’s settings were adjusted for attaining the highest sensitivity and resolution of the analytical peaks for ALC and EFB, as presented in Table 1. The optimization of the HPLC system involved the adjustment of many analytical chromatographic parameters, including the mobile phase composition and pH, as well as the characteristics of the stationary phase. The purpose of this action was to attain the highest level of distinctness and sensitivity in the chromatographic peaks of the analytes ALC and EFB, as indicated in Table 1. The mobile phase used in the experiment was an isocratic mixture comprising 45% ammonium acetate in water with a pH of 6.0 (referred to as line A), and 55% acetonitrile (referred to as line B). The mobile phase exhibited a flow rate of 0.5 mL/min. The increase in pH above 6.0, accomplished by utilizing a 10 mM ammonium acetate solution adjusted with NH4OH to a pH of 6.8, led to the manifestation of peak tailing for ALC and EFB, along with a prolonged duration of the analytical run. Upon increasing the ACN% beyond 55%, it was seen that the peaks corresponding to ALC and EFB exhibited overlap. On the other hand, a reduction in the ACN percentage led to an increase in the duration of the elution time. The ESI source was employed under positive ionization mode to enable generating parent ions. The decision was reached due to the intrinsic basicity of the nitrogen atoms found in the chemical composition of the analytes, which possess the capacity to grab protons and produce ions with a positive charge.

The MS optimization of ALC (C_30_H_34_N_4_O_2_) and EFB (C_22_H_27_ClFN_7_O_4_S) was effectively carried out using the IntelliStart^®^ software (version 4.1, SCN 805), utilizing the combined approach of direct infusion with mobile phase for ALC and EFB at 10 µg/mL. The utilization of MRM on the TQD mass analyzer was implemented in order to improve the specificity and sensitivity of the previously developed LC-MS/MS technique for the measurement of ALC and EFB. The collision cell (second quadrupole) was utilized to generate fragment ions by colliding high-purity grade argon gas with parent ions of ALC and EFB [26,27]. The measurement of the mass transition (dwell time) of ALC and EFB, precursor to daughter ions, yielded a length of 0.025 s. Table 2 presents a comprehensive list of the different dimensions of MRM, together with the mass transition of ALC and EFB (IS).

### 2.7. ALC and EFB Working Dilutions

Both ALC and EFB exhibited superior solubility in dimethyl sulfoxide (DMSO), with values of 2.0 mg/mL (4.14 mM) and 50 mg/mL (92.95 mM), respectively. Ultrasonication is required for the solubilization of both analytes. As a result, the ALC and EFB stock solutions were prepared initially with a concentration of 1 mg/mL in DMSO using the process of ultrasonication for a period of 2 min, followed by shaking, before further dilution. Three different concentrations of ALC working solutions (WKs) were prepared, namely 100, 10, and 1 µg/mL, whilst EFB was formulated at 10 µg/mL. The solutions were derived using a multi-step dilution process, commencing with the initial stock solutions of ALC and EFB, which were made at 1 mg/mL. The dilution procedure entailed the use of a solvent that closely approximated the mobile phase utilized in the HPLC equipment.

### 2.8. Construction of ALC Calibration Levels

Before performing validating tests with the established LC-MS/MS approach, the metabolic incubation matrix (HLMs) was inactivated by subjecting them to treatment with DMSO at 2% for 5 min at 50 °C. This procedure was conducted in order to inhibit the metabolic activity of the HLMs [28,29,30], hence avoiding any potential influence on the levels of ALC and EFB. To assess the validity of the proposed analytical approach, an incubation matrix was created through the dilution of 30 µL of HLMs (inactive) at a concentration of 1 mg/mL. This dilution was achieved by adding a sodium phosphate buffer (0.1 M) at pH 7.4 to reach a final volume of 1 mL. To replicate the metabolic conditions of an in vitro incubation matrix, the buffer was enhanced with a concentration of 1 mM NADPH and 3.3 mM MgCl_2_. In order to establish calibration levels for ALC, the ALC stock solution was diluted in a progressive fashion using metabolic matrix, specifically deactivated HLMs. The outcome of this procedure led to the development of seven calibration standards (CSs) at 1, 15, 50, 200, 500, 1500, and 3000 ng/mL. Furthermore, 4 quality controls (QCs) were formulated, each containing certain concentrations. These concentrations were 1 ng/mL (lower limit of quantification, LLOQ), 3 ng/mL (lower quality control, LQC), 900 ng/mL (medium quality control, MQC), and 2400 ng/mL (higher quality control, HQC). During the dilution procedure, the ratio of matrix to total volume was consistently kept above 90% in order to limit the effects of matrix dilution and replicate realistic in vitro incubation conditions. The QCs were reevaluated as unknowns, and their concentrations were calculated by employing the linear regression equation developed by the concurrent injection of analytically known concentration standards (ALC CSs). A 100 µL volume of EFB WK solution (1000 ng/mL) was used as an IS in each 1 mL of all ALC QCs and CSs.

### 2.9. Extracting the Target Analytes (ALC and EFB) from the Metabolic Matrix

The analytes ALC and EFB were extracted from the metabolic incubation (HLMs) matrix in an effective manner utilizing the protein precipitation approach. This technique included the use of ACN as both a precipitant for extraction and a quencher for metabolic processes. In the present experimental protocol, a 2 mL volume of ACN was then transferred to each of the samples containing ALC CSs and QCs. The objective of this step was to enhance the separation of ALC and EFB from the precipitated proteins. The mixture underwent agitation for a period of 5 min. Following this, centrifugation was conducted using a thermostatically controlled centrifuge, with the temperature set at 4 °C. The duration of the centrifugation process was 12 min, during which the centrifuge operated at a speed of 14,000 revolutions per minute.

To evaluate the purity and suitability of the supernatants for introduction into the LC-MS/MS instrument while maintaining the integrity of the chromatographic column, a filtration process was conducted using a 0.22 µm syringe filter. The LC-MS/MS instrument was employed to inject all filtrates, and afterwards, data were collected. Positive-control and negative-control samples were generated using the established methods mentioned earlier to confirm the lack of any interfering peaks from the components of the HLM matrix during the elution times of ALC and EFB. The positive-control sample comprised a matrix of HLMs containing EFB, while the negative-control sample comprised a matrix of HLMs without ALC and EFB. The generation of a calibration curve for ALC involved the plotting of nominal values of ALC on the *x*-axis, while the *y*-axis represented the peak area ratio of ALC to EFB. The researchers employed the linear regression equation (y = ax + b; R^2^) and validation features to assess the degree of linearity of the constructed ALC CSs.

### 2.10. Validation Features of the Proposed Analytical Method

The present analytical method (LC-MS/MS) underwent validation through the evaluation of several analytical characteristics, including accuracy, linearity, specificity, sensitivity, precision, recovery of the selected extraction approach, stability, and the incubation matrix effect. The validation process followed the analytical approach validation methods outlined by the FDA [31,32].

#### 2.10.1. Specificity

The estimation of specificity for the current LC-MS/MS method consisted of injecting six batches of negative (blank HLM matrix) controls after performing the extraction steps. The extracted samples were inserted in the LC-MS/MS instrument and evaluated for potential interference of chromatographic peaks derived from the matrix at the same retention periods as the ALC or EFB peaks. The comparison of the results was conducted by analyzing spiked samples of HLMs that included ALC and EFB. The MRM detection approach was utilized to address the carryover effect of analytes (ALC and EFB) in the triple quadrupole analyzer (TQD) system. This was demonstrated through the examination of negative-control sample HLMs without ALC and EFB.

#### 2.10.2. Linearity and Sensitivity

The verification of sensitivity and linearity of the LC-MS/MS approach was conducted by loading 12 calibration curves, each containing 7 ALC CSs, into a matrix of HLMs on a single day. Subsequently, the aforementioned samples were subjected to reanalysis in the capacity of unknowns, employing the regression equation obtained from the calibration curves established. The estimation of the LOD and LOQ was conducted in line with the procedures specified in the Pharmacopeia. The LOD and LOQ were determined by employing Equations (2) and (3), which consider the calibration curve’s slope and the standard deviation of the intercept.

(2) LOD=3.3∗SD of the interceptslope

(3) LOQ=10∗SD of the interceptslope

The assessment of linearity in the LC-MS/MS method involved the determination of the R^2^ and the utilization of the least squares method (y = ax + b).

#### 2.10.3. Accuracy and Precision

The study evaluated the accuracy and precision of the LC-MS/MS method by the injection of six sets of ALC QCs in 3 days (inter-day analysis). Additionally, twelve sets were injected in a single day (intra-day analysis). The accuracy and precision of the LC-MS/MS method were assessed by quantifying the percentage error (%E) and percentage relative SD (%RSD), correspondingly. The results were determined through the utilization of Equations (4) and (5) respectively.

(4) %Error=(average computed conc.—supposed conc.)supposed conc.∗100

(5) %RSD=SDMean

#### 2.10.4. Matrix Effect and Extraction Recovery

The study aimed to evaluate the influence of HLMs on the ionization of ALC or EFB through the formation of two separate sample cohorts. The incubation matrix of HLMs for group 1 was accompanied with the ALC LQC solution (3 ng/mL) and the EFB solution, which had a concentration of 1000 ng/mL. On the other hand, group 2 was prepared using the mobile phase as opposed to the HLM matrix. The determination of the normalized ME for the IS was carried out using Equation (6), whilst the MEs for ALC and EFB were found using Equation (7).

(6) IS normalized ME=ME of ALCME of EFB (IS)

(7) ME of ALC or EFB=mean peak area ratio group 1group 2×100

The recovery ratio of ALC extraction from the incubation matrix (HLMs) was assessed, and the influence of HLMs on the extent of ALC parent ions’ formation was evaluated by injecting 4 QCs. The validation process involved assessing the success of precipitating protein using ACN as the primary extraction approach for ALC and EFB. This was achieved by loading 6 sets of 4 QCs in incubation HLM matrix (B), and subsequently comparing them with 4 QCs arranged in the mobile phase (A). The determination of the recovery ratio for ALC and EFB was conducted by calculating the quotient of B divided by A (B/A) and afterwards multiplying the outcome by 100.

#### 2.10.5. Stability of ALC in the Stock and Working Preparations

The stability of ALC reference powder in WKs and incubation matrix (HLMs) was measured through several laboratory circumstances, including pre-analysis exposure to long- and short-term storage, three freeze–thaw cycles, and autosampler storage.

### 2.11. In Vitro Determination of ALC Metabolic Stability

The determination of hepatic intrinsic clearance (Cl_int_) and in vitro half-life (t_1/2_) of ALC required the evaluation of the residual proportion of ALC after subjecting it to an in vitro metabolic incubation with HLMs, together with the presence of nicotinamide adenine dinucleotide phosphate (NADPH) as a cofactor and MgCl_2_. The experimental protocols were executed in a systematic fashion, consisting of four discrete stages. The first stage consisted of pre-incubating a volume of ALC (1 µL) with incubation matrix (HLMs) at a temperature of 37 °C for a period of ten minutes in a water bath equipped with temperature control. During the start stage, a solution containing a concentration of 1 mM NADPH was added to all incubation samples. Subsequently, the samples were incubated at a temperature of 37 °C for a specified duration. During the third step of the experiment, a solution containing 100 µL of EFB at a concentration of 1000 ng/mL was introduced before the ACN addition, which was used as a stopping mediator. The purpose of this action was to mitigate potential disruptions to the concentration of the internal standard (IS) resulting from the activity of metabolic enzymes. In the fourth step, the metabolic process was terminated at certain time points (0, 2.5, 7.5, 15, 20, 30, 40, 50, 60, and 70 min). To achieve this, 2 mL of ACN was introduced to all incubates. The inclusion of ACN in this process aimed to inhibit metabolic pathways and facilitate precipitating of remaining proteins. This phase serves as the first step in the extraction method for ALC and EFB, as defined in Section 2.9. A sample of ALC with HLMs (negative control), in the absence of NADPH, was generated following the same procedures as previously. The purpose of this study was to confirm that there were no incubation characteristics or matrix effects that could impact the measured concentration of ALC in the experimental investigations.

The residual concentration of the ALC was estimated by employing the regression equation of the concurrently loaded ALC calibration standards (CSs). The ALC metabolic stability curve was graphed by scheming the selected time intervals (*x*-axis) spanning from 0 min to 70 min against the ALC% concentration that persisted compared to the starting concentration (100%) at time zero (*y*-axis). Following this, the initial portion of the curve ranging from 0 to 40 min, which displayed a linear correlation, was selected in order to generate an extra curve using a natural logarithmic modification. The procedure consisted of graphing the time points of metabolic incubation, ranging from 0 to 40 min, against the natural logarithm (ln) of ALC concentrations. The slope of the established curve was utilized to estimate the in vitro t_1/2_ through the utilization of the formula in vitro t_1/2_ = ln2/slope. The ALC Cl_int_ (mL/min/Kg) was calculated [33] by employing the HLM matrix (45 mg/gm) of liver tissue (26 g/Kg of body weight) (Equation (8)) [34].

(8) $${Cl}_{int,}=0693\times \frac{1}{t_{½}(min.)}\times \frac{mL\;incubation}{mg\;protein}\times \frac{mg\;microsomal\;proteins}{g\;of\;liver\;weight}\times \frac{g\;liver}{Kg\;b.w.}$$

## 3. Results

### 3.1. In Silico Assessment of ALC Metabolic Stability

**Figure 2:**
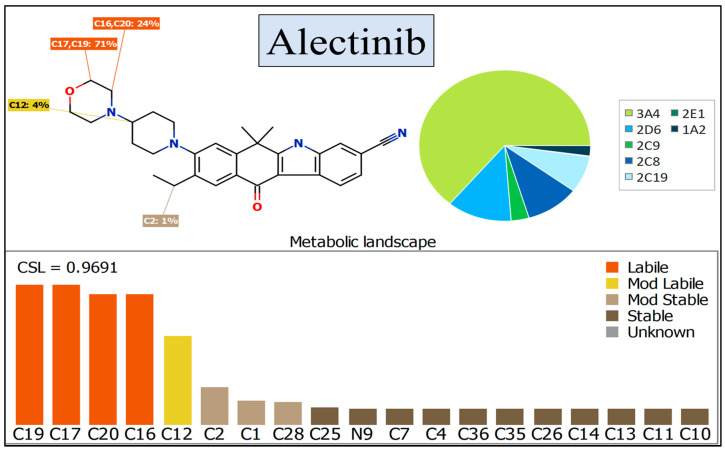
The composite site lability (CSL) value of 0.9691 indicates that ALC exhibits a moderate level of lability in terms of its metabolism. The outcomes were assessed utilizing the P450 program of StarDrop software.

The profile of ALC metabolism was employed to demonstrate the susceptibility of active sites in ALC to metabolism by the CYP3A4 enzyme, as depicted in the pie chart [35,36,37]. The composite site lability (CSL) value of ALC was estimated to be 0.9691, revealing a moderate level of metabolic lability of ALC (Figure 2). This finding aligns with the results obtained from the practical assessment of ALC’s metabolic stability using the LC-MS/MS method. The metabolic lability of ALC was found to be 24% for carbon atoms C16 and C20 and 71% for carbon atoms C17 and C19, indicating a significant degree of lability at the morpholine group. Additionally, a metabolic lability of 12% was observed for carbon atom C12, indicating a moderate degree of lability at the piperidine ring. Based on the observed results, it can be deduced that the inclusion of the morpholine group had a notable impact on the metabolic stability of ALC (as illustrated in Figure 2; CSL: 0.9691, suggesting a moderate level of vulnerability to metabolic pathways). The aforementioned outcomes are consistent with the outcomes derived from the ensuing in vitro practical incubations, which will be further elaborated upon in coming sections.

### 3.2. In Silico DEREK Module Prediction of ALC Toxic Alerts

**Figure 3:**
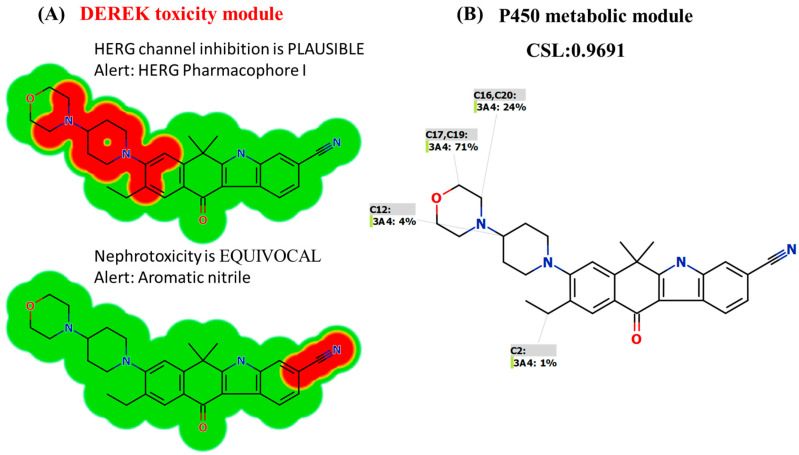
Structural alerts of ALC using DEREK software highlighted in red (**A**). P450 metabolic stability of ALC CSL at 0.9691 indicating the moderate clearance characteristic of ALC (**B**).

The toxicity evaluation of ALC was conducted utilizing the DEREK software in silico (Figure 3A). The compound ALC exhibits structural warnings, as illustrated in Figure 3. These alerts are responsible for the hypothesized effects, namely HERG channel inhibition (plausible) due to HERG pharmacophore I and nephrotoxicity (equivocal) due to aromatic nitrile. The proposed side effect (HERG channel inhibition) is related to morpholine and piperidine rings that are connected together (HERG pharmacophore I that matched with the previous results of in silico metabolic stability) (Figure 3B). Minor structural alterations to the bioactive groups (morpholine and piperidine rings) or substitution of the group in drug design has the potential to enhance the metabolic stability and safety profile of novel derivatives in comparison to ALC (Figure 3).

### 3.3. In Silico ADME Profile

**Figure 4:**
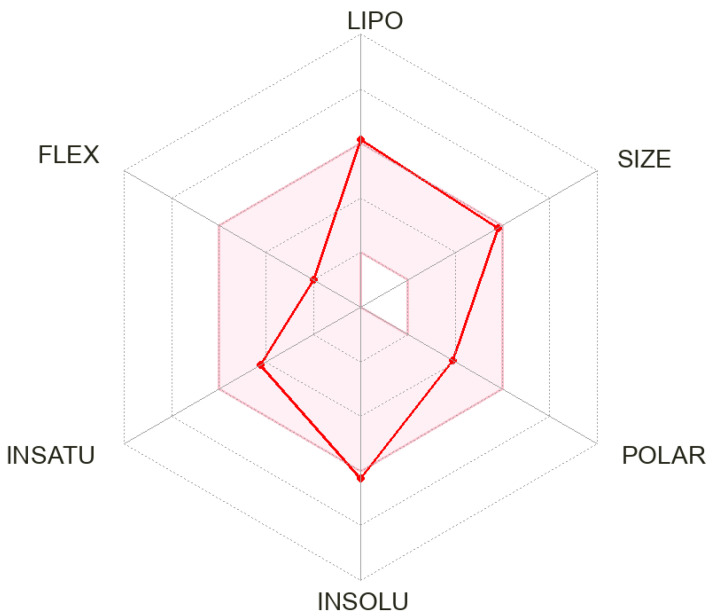
The ADME radar chart of ALC obtained from SwissADME software indicating the good ADME characteristics of ALC that fall within the suitable range for a pharmacological drug.

An assessment of the druglikeness of ALC was conducted through an analysis of its ADME characteristics. According to the log *p* value predicted by the SwissADME software (http://www.swissadme.ch/ (accessed on 20 May 2023)), it was observed that the solubility of ALC in water was found to be low (LogS = −6.25). Furthermore, the anticipated pharmacokinetic parameter for absorption in the gastrointestinal tract is significantly elevated with permeability across the blood–brain barrier. The bioavailability score is reported to be 0.55. The compound ALC is anticipated to function as an inhibitor for some cytochrome P450 enzymes (namely CYP2C19 and CYP2C9) as well as P-glycoprotein, which is a substrate. It is proposed that ALC is not an inhibitor for some cytochrome P450 enzymes (namely CYP1A2, CYP3A4, and CYP2D6). The LogKp value for skin permeability prediction is −5.52 cm/s. In terms of drug similarity, it adheres to the Lipinski rule. Additionally, it violates the Ghose rule with two violations: MW > 480 and MR > 130 and it also violates the Muegge rule (one violation: XLOGP3 > 5). Figure 4 displays the ADME radar chart for ALC, while Table 3 provides the corresponding statistics.

### 3.4. LC-MS/MS Method

**Figure 5:**
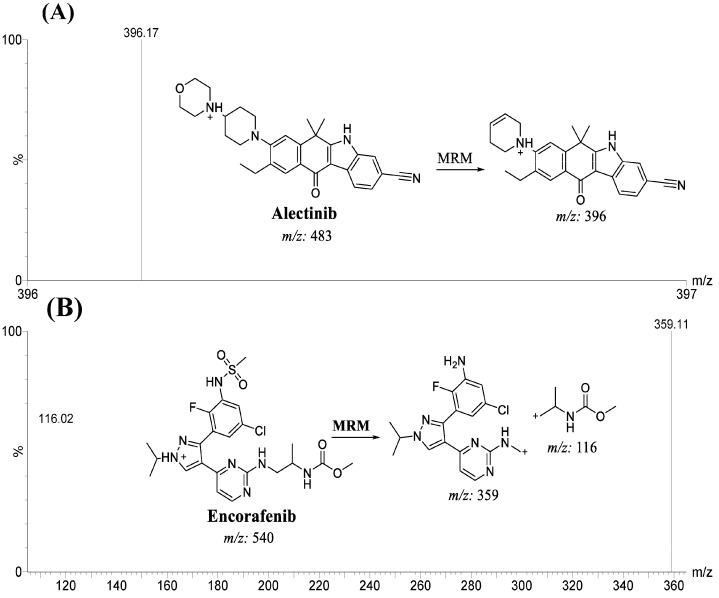
MRM mass spectrum of ALC [M + H]^+^ showing one mass transition from *m*/*z* 483 to *m*/*z* 396 (**A**) and EFB [M + H]^+^ showing two mass transitions from *m*/*z* 540 to *m*/*z* 359 and from *m*/*z* 540 to *m*/*z* 116 (**B**). The proposed collision-induced dissociation (the collision cell) patterns are exhibited.

Multiple stationary phases, involving HILIC columns, were assessed; however, both ALC and EFB were not subjected to chromatographic separation. Nevertheless, the favorable results were attained while utilizing the reversed C8 column. The application of a C18 column in the LC-MS/MS method for the separation of ALC and EFB leads to the retention of both analytes. However, this retention is associated with insufficient chromatographic peak separation, peak tailing, and a prolonged retention period. Satisfactory results were achieved when employing an Eclipse plus-C8 column with certain dimensions, including an ID of 2.1 mm, a PS of 3.5 μm, and an L of 50 mm. These results were observed in relation to the quality of chromatographic peak shape as well as the duration of retention time. The LC-MS/MS technique being evaluated utilized mobile phase running in an isocratic system with a 0.5 mL/min flow rate for a period of two minutes to separate ALC and EFB. The linear relationship of the calibration curve for ALC was seen within the range of 1 to 3000 ng/mL. To improve the LC-MS/MS technique sensitivity, the MRM mode of the TQD system was utilized for precise mass estimation and identification of ALC and EFB. The selection of this methodology was made in order to minimize any possible disruption caused by the components present in the matrix of human liver microsomes (HLMs), as illustrated in Figure 5.

**Figure 6:**
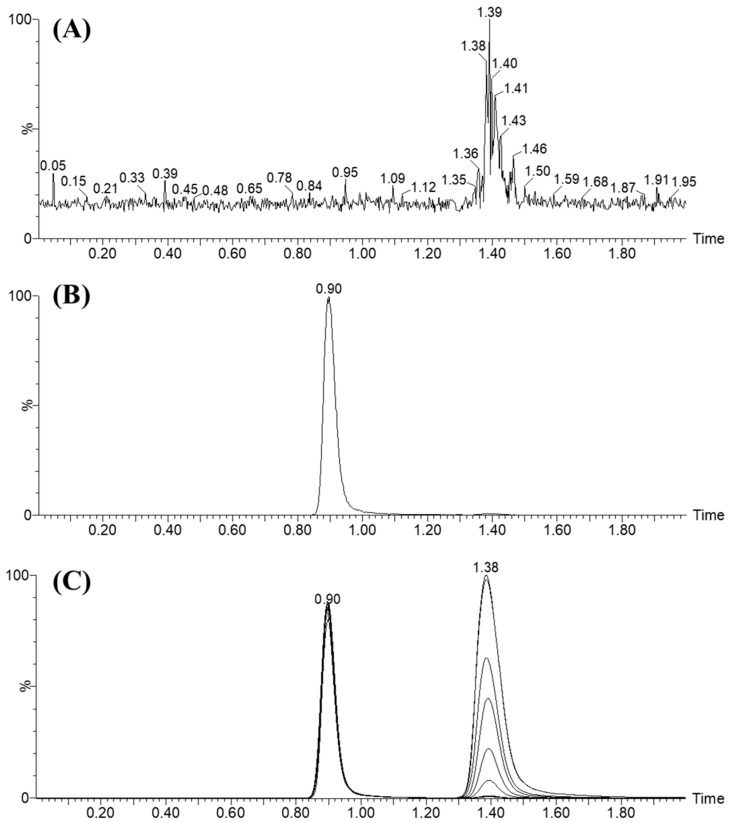
Panel (**A**) displays the negative-control sample consisting of HLM matrix, which exhibits no interference at the retention times of alectinib (ALC) and encorafenib (EFB). Panel (**B**) presents the MRM chromatogram of the positive-control sample, which contains HLMs with EFB at a concentration of 1000 ng/mL. Panel (**C**) showcases the overlaid MRM charts of ALC standards, including CSs and quality controls (QCs) at various concentrations ranging from 1 to 3000 ng/mL. The ALC peak is observed at 1.38 min, while the EFB peak (at a concentration of 1000 ng/mL) appears at 0.9 min, indicating a fast LC-MS/MS method.

The utilization of EFB as the selected internal standard (IS) for the quantification of ALC in the LC-MS/MS approach was influenced by three parameters. Initially, the ALC and EFB were effectively isolated using the protein precipitation technique, resulting in notable yields of 100.27 ± 2.65 (RSD: 2.64) and 98.36 ± 3.75% (RSD: 3.81%), respectively. Furthermore, it was observed that the chromatographic peaks corresponding to EFB (0.9 min) and ALC (1.38 min) were effectively resolved and eluted within a 2 min timeframe. This outcome serves as evidence of the rapidity and effectiveness of the developed LC-MS/MS technique, which notably necessitates just a minimal quantity of ACN, thus aligning with the principles of green chemistry. Moreover, the administration of the ALC and EFB therapies was varied among the patients. Hence, the suggested LC-MS/MS technique might be utilized for conducting TDM and pharmacokinetic experiments of ALC. The MRM chromatograms of the positive (Figure 6A) and negative controls (Figure 6B) did not exhibit any indications of ALC carry-over. Figure 6C exhibits the superimposed MRM mass chromatograms for the ALC calibration levels, encompassing both CLs and QCs (1, 3, 15, 50, 200, 500, 900, 2400, and 3000 ng/mL), as well as EFB 1000 ng/mL.

### 3.5. Validation Parameters of the Current LC-MS/MS Method

#### 3.5.1. Specificity

The specificity of the current LC-MS/MS technique was verified by the discrete chromatographic separation of ALC and EFB peaks, as exhibited in Figure 6. Additionally, it was detected that there was no discernible interference between the chromatographic peaks of ALC and EFB originating from the matrix constituents of HLMs. The MRM chromatograms of the control samples did not demonstrate any discernible carry-over effect of the analyte ALC.

#### 3.5.2. Linearity and Sensitivity

**Figure 7:**
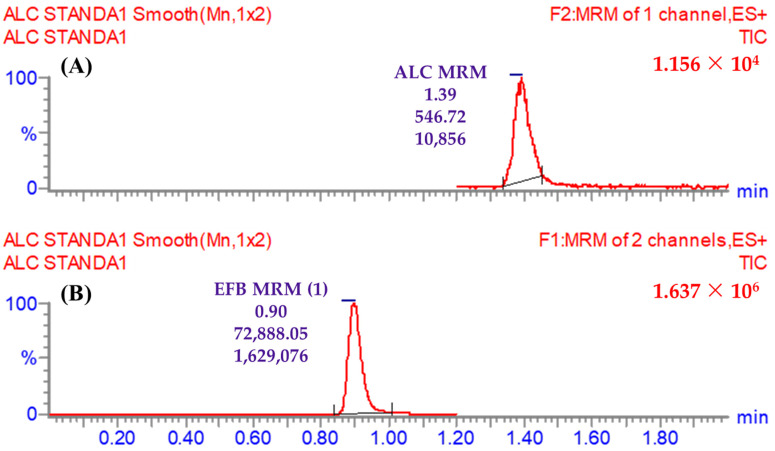
(**A**) ALC chromatographic peak (1.39 min) at 1 ng/mL (LLOQ) indicating the sensitivity of the proposed LC-MS/MS method; (**B**) EFB (IS) analytical peak (0.9 min) at 1000 ng/mL. The LLOQ can be detected very easily as seen in the figure with a height of 546.72 and peak area of 10,856 that reveals the high sensitivity of the established LC-MS/MS approach.

The statistical verification of the LC-MS/MS technique linearity was achieved throughout the practical range of 1 ng/mL to 3000 ng/mL, as evidenced by the linear equation y = 1.011x + 8.342 and a coefficient of determination (R^2^) value of 0.9959. The accomplishment of this task involved the incorporation of seven ALC CSs into the HLM matrix, followed by their subsequent recalculation as variables of unknown value. The utilization of the reciprocal function (1/x) was included in the development of the ALC calibration curve in order to account for the wide span of concentrations (ranging from 1 to 3000 ng/mL). Table 4 displays the coefficient of variations (CVs), also referred to as relative standard deviations (RSDs), for the six repeated measurements. It is observed that all the RSDs are below 3.45%. The values for the limits of detection (LOD) and quantification (LOQ) were established as 0.27 ng/mL and 0.82 ng/mL, respectively, as depicted in Figure 7.

#### 3.5.3. Precision and Accuracy

The accuracy and precision of the current LC-MS/MS approach described in this study were assessed by conducting experiments on multiple days. Specifically, 12 sets of samples, each containing four quality control (QC) samples, were analyzed on a single day (intra-day validation). Additionally, six sets of samples, each containing four QC samples, were analyzed over the course of three days (inter-day validation). The findings fell in the permitted range as specified by the validation rules of the FDA [38]. The precision and accuracy of the LC-MS/MS methodology presented in this study were evaluated for both inter-day and intra-day measurements. The inter-day accuracy ranged from −2.56% to 3.45%, while the inter-day precision ranged from −3.78% to 4.33% (Table 5).

#### 3.5.4. Extraction and Recovery of ALC in the Proposed LC-MS/MS Method

The efficacy of the certain protein precipitation technique for extracting ALC and EFB was assessed by loading six replicates (including four QCs) into incubation HLM matrix, and subsequently comparing the acquired outcomes with quality controls made in the mobile phase. The results indicated a significant recovery ratio for ALC, with a value of 100.27 ± 2.65% and an RSD of less than 2.64%. In a similar vein, the recovery ratio of extracting EFB was determined to be 98.36 ± 3.75%, exhibiting an RSD of less than 3.81%. The investigation of two sets of injection samples of HLM matrices has yielded results indicating that there is no statistically significant impact on the generation of parent ions, specifically ALC or EFB. Group set 1 was enhanced with LQC (3 ng/mL) and EFB (1000 ng/mL), whereas group set 2 was generated using the mobile phase. HLMs that included ALC and EFB had an ME of 101.27 ± 1.68% and 99.76 ± 3.26%, correspondingly. The normalized ME of the IS was 1.01, which is in the suitable range specified by the regulations established by the FDA. The results of the investigation suggest which the HLM matrix does not show a statistically significant effect on the ion formation of EFB or ALC.

#### 3.5.5. ALC was Stable in the Stock and Working Preparations

The stability of the ALC in DMSO and its stability in HLMs were evaluated, and it was observed that the ALC demonstrated acceptable stability during storage in DMSO at −80 °C for a period of 28 days. The study determined that the %RSD for all ALC samples was below 3.91% across diverse storage settings (Table 6) and no substantial reduction in the ALC concentration was found. The results of the study clearly indicated the absolute stability of ALC.

#### 3.5.6. An Assessment of the Environmental Sustainability of the Current LC-MS/MS Technology Utilizing the AGREE Program

**Figure 8:**
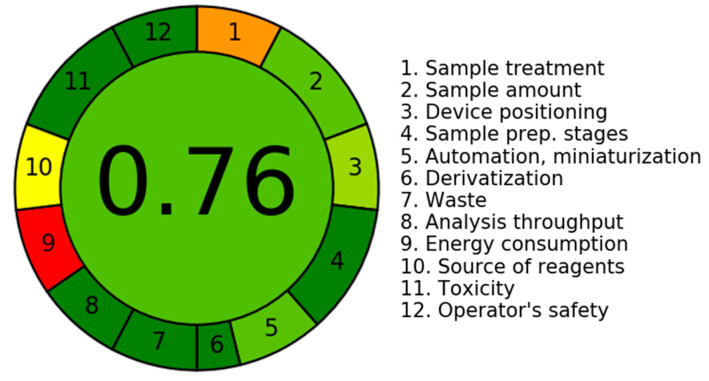
The results are presented in a circular drawing that showcases a diverse spectrum of hues, ranging from dark green (indicating the maximum degree of greenness) to red (revealing a lack of greenness). The colors described above are linked to twelve separate attributes, as illustrated in the accompanying visual depiction. The GAC score was 0.76, as seen in the middle of the circle, that indicated the greenness of the current LC-MS/MS approach.

The assessment of the environmental sustainability, also referred to as the level of “greenness”, of the current LC-MS/MS method, was carried out using the in silico program known as AGREE. The program incorporates all twelve criteria that have been defined by the GAC community [12]. The software utilizes a range of weights between 0.0 and 1.0 to give values to different properties of the GAC system. This process leads to the development of analytical scales that effectively measure the degree of environmental sustainability. The results are visually represented by a circular diagram that includes a vast range of colors, covering from dark green to red, which symbolize twelve separate attributes. Figure 8 illustrates the present employment of LC-MS/MS technology with regard to its environmental sustainability. The scores associated with each of the twelve qualities were recorded and are presented in Table 7. The study of many aspects pertaining to the current methodology resulted in a score of 0.76 that indicated the greenness of the present LC-MS/MS methodology.

### 3.6. In Vitro Incubations of ALC with Metabolic HLM Matrix

**Figure 9:**
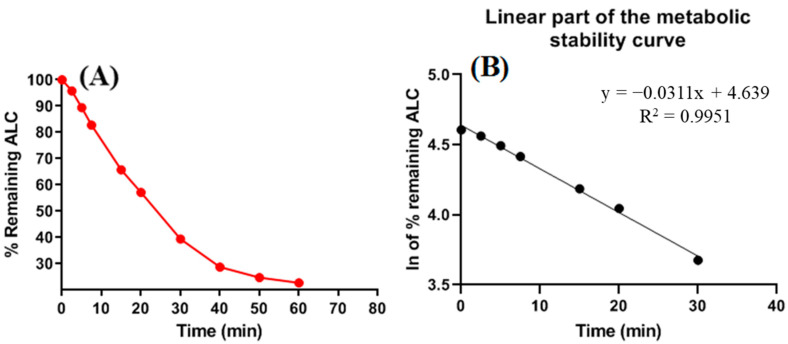
The metabolic stability curve of ALC in the incubation matrix of HLMs was analyzed from 0.0 to 60 min. (**A**). Additionally, the linear section of the logarithmic (ln) calibration curve was examined, showing the regression equation (y = −0.0311x + 4.639; R^2^ = 0.9951) revealing a slope of −0.0311 that was utilized in calculation of t_1/2_ (22.28 min) and Cl_int_ (36.37 mL/min/kg) (**B**).

The negative-control sample did not demonstrate a significant decrease in the concentration of ALC. In order to assess the metabolic stability of the target analyte (ALC) in an in vitro environment, a concentration of 1 µM/mL was employed. The concentration was deliberately chosen to be below the Michaelis–Menten constant to ensure the linearity among the rate of ALC metabolism and length of the incubation. To address the issue of non-specific protein binding, HLMs (1 mg protein/mL) were employed. Figure 9a illustrates the establishment of the initial ALC stability curve through the plotting of time points for metabolic reaction stopping (ranging from 0 to 70 min) on the *x*-axis and the representation of the residual ratio of ALC on the *y*-axis. The decision was made to choose a linear segment of the curve that encompasses the time interval between 0 and 40 min. The purpose of this option was to create an additional curve that is specifically focused on the range of 0 to 40 min. Figure 9b displays the second curve, which represents the natural logarithm of the residual ratio of ALC in relation to the chosen time interval. The metabolic rate (slope) of ALC was found to be 0.0311 (y = −0.0311x + 4.639, r^2^ = 0.9951) as shown in Table 8. The determination of the in vitro t_1/2_ can be achieved through the division of the natural logarithm of 2 by the slope. As a result, the in vitro t_1/2_ was found to be 22.28 min. The ALC Cl_int_ (clearance) was calculated to be 36.37 mL/min/kg. Agreeing with the scaling technique developed by McNaney et al. [33], it can be deduced that ALC falls under the category of an intermediate clearance medication. Various software applications, including Cloe PK and modeling software, can be utilized to evaluate the in vivo pharmacokinetics of ALC [39].

## 4. Discussions

A sensitive, ecofriendly, and selective LC-MS/MS approach was developed for estimation of ALC in the HLM matrix. The established analytical approach was validated following the FDA guidelines. The LC-MS/MS approach that was recommended exhibited accuracy and precision levels for both inter-day and intra-day measurements. Specifically, the accuracy values ranged from −2.56% to 3.45%, while the precision values ranged from −3.78% to 4.33%. The sensitivity of the established approach was proved by its ability to adhere to an LLOQ of 0.82 ng/mL. ALC demonstrated good stability during storage in DMSO at −80 °C for a period of 28 days. No substantial decrease in the ALC concentration was found following periods of long-term storage, autosampler storage, short-term storage, and three freeze–thaw cycles that matched the reported data of the stability of ALC in biological fluids including cerebrospinal fluid at room temperature for up to two days. Also, ALC was reported to be stable in the autosampler for up to 23 h and during three freeze–thaw cycles [26].

The score of AGREE (0.76) for the current analytical approach serves as a quantitative measure for evaluating the degree of environmental sustainability achieved through the implementation of the LC-MS/MS technique. It is vital to recognize that a greater score, approaching 1.0, signifies an elevated level of environmental sustainability inside the evaluation process. The LC-MS/MS technology exhibits a notable degree of environmental sustainability, as evidenced by eco-scale scores that span from 0.75 to 1.00.

The developed LC-MS/MS was applied for metabolic stability assessment of ALC in HLMs. The half-life (t_1/2_) and intrinsic clearance (Cl_int_) of ALC were determined to be 22.28 min and 36.37 mL/min/kg, correspondingly, that indicated a moderate extraction ratio. The CSL value of ALC was 0.9691 that indicated a moderate level of metabolic lability of ALC. The morpholine ring in the chemical structure of ALC had a major impact on the metabolic stability of ALC. These in silico results are consistent with the data derived from the ensuing in vitro metabolic experiments. The P450 and DEREK modules of the StarDrop software package were used for screening of the metabolic stability and identification of hazardous alarms within the chemical structure of ALC. The morpholine ring and piperidine rings are cyclic tertiary amines that were reported in many drugs to be responsible for bioactivation to reactive intermediates that may be responsible for side effects of ALC [40,41]. The metabolic stability and safety properties of newly created derivatives can be enhanced by making modest adjustments to the morpholine and piperidine rings or by substituting the substituent, as per computational software.

The data generated from the practical experiments and various in silico software were found to be aligned with the literature and the official reports of ALC in the following way: ALC exhibited moderate clearance that supports the regular dosage regimen of ALC (starting dose: 600 mg PO BID) followed by sequential dose reduction to first dose reduction: 450 mg PO BID then 300 mg PO BID and finally discontinue if patients are unable to tolerate 300 mg PO BID. BBB permeant is expected by ADME software as reported in the literature [42]. Solubility: according to ADME software, ALC exhibits poor water solubility that matched the reported solubility in DMSO on the MedChem Express company (Monmouth Junction, NJ, USA) website (https://www.medchemexpress.com/CH5424802.html (accessed on 20 May 2023).

The primary factors contributing to the failure of drug discovery compounds in clinical settings are the absence of therapeutic effectiveness and the presence of harmful effects. In order to mitigate the occurrence of late-stage failures within the drug discovery process, it is imperative to accurately assess the likelihood of adverse effects and probable toxicity at an early stage. Cardiotoxicity frequently manifests as a consequence of a compound’s blockage of the HERG channel, which plays a crucial role in regulating the flow of potassium cations [43]. Symptomatic bradycardia is reported for ALC in some patients that results in withholding ALC until recovery to asymptomatic bradycardia or to a heart rate ≥ 60 bpm. The expected side effects by DEREK software are nephrotoxicity and ALC should be temporarily stopped until serum creatinine recovers to ≤1.5× ULN, then resumed at reduced dose or permanently discontinued (Grade 4) [44].

From the previously mentioned details, the current approach could be used successfully in saving time and resources in the early stage of drug development and could also help in designing safer derivatives of the current approved drugs through identifying of the different structural alerts in the chemical structure of the target drugs. The utilization of different in silico software in addition to practical experiments was performed in a stepwise protocol, saving money and resources. This research design could be applied to screen and refine promising molecules in the process of drug design. This approach could also help in designing new derivatives of the drug that might retain the same biological activity by carrying out targeted structural modifications after finding the structural alerts inside the chemical structure of a drug.

## 5. Conclusions

**Figure 10:**
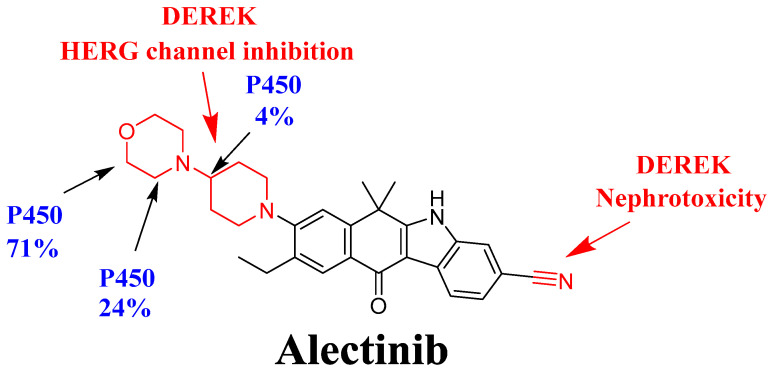
ALC metabolic lability curve (blue) using P450 software (version 6.6) and ALC DEREK toxicity predictions (red) indicating that morpholine and piperidine rings are responsible for the metabolic instability of ALC.

The current investigation focused on developing and verifying an LC-MS/MS technique for quantifying ALC in the metabolic matrix of HLMs. The aforementioned technique was subsequently utilized to assess the metabolic stability of ALC. The LC-MS/MS method employed in this study exhibited desirable selectivity, sensitivity, and effective recovery of ALC and EFB from the incubation matrix (HLMs) using the selected precipitation of proteins as an extraction technique. The LC-MS/MS technique used in this study demonstrates environmental sustainability through the implementation of a low flow rate of 0.5 mL/min, reduced utilization of ACN, and a notably shortened elution time of 2 min. Following an assessment of the greenness utilizing the AGREE program, it can be inferred that the present LC-MS/MS technique exhibits favorable environmental characteristics and may be more appropriate for routine ALC analysis without detrimental impacts on the surrounding ecosystem. The verification process involved comparing the results produced from the in silico metabolic P450 model utilized in the software StarDrop with the outcomes of in vitro incubation experiments performed using HLMs. The findings related to metabolic stability, notably a t_1/2_ of 22.28 min and a moderate extraction ratio of 36.37 mL/min/kg, suggest that ALC can be categorized as a pharmaceutical exhibiting a moderate extraction ratio. Henceforth, it is proposed that giving ALC to patients will not lead to the buildup of dosages within the body. Future research endeavors may encompass the employment of computational software tools in conjunction with laboratory studies to explore and advance the development of novel medications that exhibit improved metabolic stability. The comparable outcomes found from in vitro metabolic incubation tests and in silico program analysis of ALC prove the efficacy of the utilized software in reducing both the amount of time and resources required. Based on the results obtained from computational P450 metabolic and DEREK software analyses, it is suggested that introducing slight structural alterations to the morpholine and piperidine rings or substituting the group during the drug design process might present an opportunity to enhance the metabolic stability and safety profile of novel derivatives that might not influence the pharmacological activity (in comparison to ALC) or could be considered during the progression of novel pharmaceutical development, specifically in augmenting metabolic stability (Figure 10).

## Figures and Tables

**Table 1:** LC-MS/MS instrumental parameters.

LC (H10UPH)		MS/MS (QBB1203)	
Binary mobile phase	45% line A: ammonium acetate in H_2_O (pH: 6.0)	ESI	Positive ESI source
	55% line B: ACN		Cone gas: 100 L/H flow rate
	0.5 mL/min flow rate		The voltage of RF lens: 0.1 (V)
	Injection volume: 5.0 μL		The voltage of extractor: 3.0 (V)
Eclipse plus-C8 column	50.0 mm long		Capillary voltage: 4 KV
	2.1 mm i.d.		Nitrogen (drying gas; 350 °C) at 100 L/h
	3.5 μm	Mode of detection	MRM
	23.0 ± 1.0 °C	Collision cell	Argon gas (0.14 mL/min)

**Table 2:** MRM-optimized parameters for the estimation of ALC and EFB (IS).

	Time	Retention Time	MRM Mass Transitions	
Time segments	1.2 to 2.0 min	ALC (1.39 min)	One mass transition (*m*/*z*)	483 → 396 (CE ^a^: 18 and CV ^b^: 50)
	0.0 to 1.2 min	EFB (IS; 0.9 min)	First mass transition (*m*/*z*)	540 → 359 (CE: 32 and CV: 56)
			Second mass transition (*m*/*z*)	540 → 116 (CE: 36 and CV: 56)

^a^ Collision energy, ^b^ cone voltage.

**Table 3:** ADME properties of ALC screened by SwissADME software.

Physicochemical Properties		Water Solubility	
Formula	C_30_H_34_N_4_O_2_	LogS (ESOL)	−6.25
Molecular weight	482.62 g/mol	Solubility	2.71 × 10^−4^ mg/mL; 5.62 × 10^−7^ mol/L
Num. arom. heavy atoms	15	Class	Poorly soluble
Num. heavy atoms	36	LogS (Ali)	−6.52
Num. rotatable bonds	3	Solubility	1.46 × 10^−4^ mg/mL; 3.03 × 10^−7^ mol/L
Fraction Csp3	0.47	Class	Poorly soluble
Num. H-bond donors	1	Solubility	1.91 × 10^−6^ mg/mL; 3.96 × 10^−9^ mol/L
Num. H-bond acceptors	4	Class	Poorly soluble
TPSA	72.36 Å^2^	**Medicinal Chemistry**	
Molar refractivity	149.63	PAINS	0 alert
**Lipophilicity**		Leadlikeness	No; 2 violations: MW > 350, XLOGP3 > 3.5
LogPo/w (XLOGP3)	5.25	Brenk	0 alert
LogPo/w (MLOGP)	2.39	Synthetic accessibility	3.92
LogPo/w (iLOGP)	4.08	**Pharmacokinetics**	
LogPo/w (SILICOS-IT)	5.93	GI absorption	High
LogPo/w (WLOGP)	4.01	P-gp substrate	Yes
Consensus LogPo/w	4.33	BBB permeant	Yes
**Druglikeness**		CYP2C9 inhibitor	Yes
Ghose	No; 2 violations: MW > 480, MR > 130	CYP1A2 inhibitor	No
Muegge	No; 1 violation: XLOGP3 > 5	CYP2C19 inhibitor	Yes
Egan	Yes	CYP2D6 inhibitor	No
Veber	Yes	CYP3A4 inhibitor	No
Lipinski	Yes; 0 violation	LogKp (skin permeation)	−5.52 cm/s
Bioavailability score	0.55		

**Table 4:** Summary of recalculation results of six replicates (CSs) of alectinib (ALC).

ALC (ng/mL)	Mean	SD	RSD (%)	Accuracy (%)	Recovery
1.0	1.02	0.04	3.45	1.67	101.67
15.0	14.76	0.09	0.58	−1.60	98.40
50.0	52.57	0.87	1.66	5.13	105.13
200.0	194.46	1.48	0.76	−2.77	97.23
500.0	491.42	5.14	1.05	−1.72	98.28
1500.0	1511.66	16.56	1.10	0.78	100.78
3000.0	3011.51	14.12	0.47	0.38	100.38
% Recovery					100.27 ± 2.65

**Table 5:** Accuracy and precision of the proposed LC-MS/MS method.

ALC (ng/mL)	Intra-Day (Twelve Groups on One Day)				Inter-Day (Six Groups in Three Days)			
QCs	1	3	900	2400	1	3	900	2400
Mean	1.02	2.92	900.33	2388.72	2.89	892.77	2389.93	1.04
SD	0.04	0.03	11.01	15.82	0.05	5.19	3.70	0.01
Precision (%RSD)	3.45	1.05	1.22	0.66	1.64	0.58	0.15	1.11
% Accuracy	1.67	−2.56	0.04	−0.47	−3.78	−0.80	−0.42	4.33
Recovery (%)	101.67	97.44	100.04	99.53	96.22	99.20	99.58	104.33

**Table 6:** Summary of the stability data of ALC.

Stability Features	3.0	2400.0	3.0	2400.0	3.0	2400.0	3.0	2400.0
	Mean		SD		RSD (%)		Accuracy (%)	
Freeze–Thaw Stability (three cycles at −80 °C)	3.07	2421.62	0.04	5.17	1.42	0.21	2.33	0.90
Autosampler Stability (24 h at 15 °C)	3.05	2411.13	0.12	15.06	3.91	0.62	1.78	0.46
Long-Term Stability (−80 °C for 28 d)	3.01	2418.62	0.03	10.85	1.01	0.45	0.44	0.78
Short-Term Stability (4 h at room temperature)	2.93	2403.33	0.04	5.66	1.23	0.24	−2.33	0.14

**Table 7:** The report sheet for the current LC-MS/MS approach has been specifically developed to assess the environmental sustainability of the process. This is achieved by assigning individual ratings based on the principles of GAC. The scores are graphically presented in a circular manner, utilizing a diverse spectrum of colors ranging from red (0.0, denoting the complete lack of greenness) to dark green (1.0, marking the highest level of greenness). The colors stated above are linked to twelve distinct characteristics, which are enumerated in the table.

Criteria	Score	Weight
1. It is recommended to utilize direct analytical procedures in order to minimize the necessity for sample treatment.	0.3	2
2. The aims of this investigation are to attain a limited sample size and reduce the quantity of samples.	0.75	3
3. Ideally, it is recommended to perform measurements inside their original contextual environment.	0.66	
4. The incorporation of analytical measures with operational tactics has been observed to decrease in reagent depletion and produce energy preservation.	1.0	2
5. It is advisable to consider the adoption of automated and streamlined processes.	0.75	3
6. It is recommended to abstain from utilizing derivatization processes.	1.0	2
7. The minimization of the production of a significant volume of analytical surplus and the adoption of efficient solutions for its proper disposal are of paramount significance.	1.0	1
8. The inclination is towards employing multi-analyte or multi-parameter methodologies rather than relying solely on single-analyte approaches.	1.0	2
9. The prioritization of endeavors aimed at minimizing energy use is of paramount significance.	0.0	2
10. It is recommended to give priority to the utilization of reagents obtained from environmentally friendly sources.	0.5	2
11. The prioritizing of the removal or replacement of hazardous substances is of utmost importance.	1.0	3
12. There exists a significant imperative to augment the safety practices for operators.	1.0	3

**Table 8:** Metabolic stability of alectinib (ALC).

Time (Min)	Mean ^a^	X ^b^	LN X	Linearity Characteristics
0.00	473.85	100.00	4.61	Regression line equation: y = −0.0311x + 4.639
2.50	453.85	95.78	4.56	
5.00	423.86	89.45	4.49	R^2^ = 0.9951
7.50	392.11	82.75	4.42	
15.00	311.51	65.74	4.19	Slope: −0.0311
20.00	270.81	57.15	4.05	
30.00	186.93	39.45	3.68	t_1/2_: 22.28 min and
40.00	136.23	28.75	3.36	Cl_int_: 36.37 mL/min/kg
50.00	117.28	24.75	3.21	
60.00	107.94	22.78	3.13	

^a^ Mean of three replicates, ^b^ X: Mean of the % remaining of ALC in three replicates.

## Data Availability

All data are available within the manuscript.
